# Mixed-litter effects of fresh leaf semi-decomposed litter and fine root on soil enzyme activity and microbial community in an evergreen broadleaf karst forest in southwest China

**DOI:** 10.3389/fpls.2022.1065807

**Published:** 2022-12-09

**Authors:** Bing Mao, Tingting Cui, Tongqing Su, Qiangsheng Xu, Feng Lu, Hongxin Su, Jianbing Zhang, Shuangshuang Xiao

**Affiliations:** ^1^ Key Laboratory of Environment Change and Resources Use in Beibu Gulf, Ministry of Education, Guangxi Key Laboratory of Earth Surface Processes and Intelligent Simulation, Nanning Normal University, Nanning, China; ^2^ Institute of Ecology, College of Urban and Environmental Sciences and Key Laboratory for Earth Surface Processes of the Ministry of Education, Peking University, Beijing, China; ^3^ Laibin Jinxiu Dayaoshan Forest Ecosystem Observation and Research Station of Guangxi, Laibin, China

**Keywords:** litter mixing, litter type, litter quality, richness, microbial diversity

## Abstract

Litter decomposition is the main process that affects nutrient cycling and carbon budgets in mixed forests. However, knowledge of the response of the soil microbial processes to the mixed-litter decomposition of fresh leaf, semi-decomposed leaf and fine root is limited. Thus, a laboratory microcosm experiment was performed to explore the mixed-litter effects of fresh leaf, semi-decomposed leaf and fine root on the soil enzyme activity and microbial community in an evergreen broadleaf karst forest in Southwest China. Fresh leaf litter, semi-decomposed litter and fine root in the *Parakmeria nitida* and *Dayaoshania cotinifolia* forests, which are unique protective species and dominant species in the evergreen broadleaf forest, were decomposed alone and in all possible combinations, respectively. Our results showed that the mass loss of fresh leaf litter in three mixed-litter treatment was significantly higher than that in two mixed-litter treatment in the *P. nitida* and *D. cotinifolia* forests. Mass loss of fine root in the single litter treatment was significantly lower in the *P. nitida* forest and higher in the *D. cotinifolia* forest compared to that in the other litter treatments. There were insignificant differences in the activities of β-glucosidase (BG) and leucine aminopeptidase (LAP) between control and mixed-litter treatment in the *P. nitida* forest and between control and single litter treatment in the *D. cotinifolia* forest. The N-acetyl-β-D-glucosaminidase (NAG) activity was significantly increased by the single litter decomposition of fresh leaf and fine root and three mixed-litter decomposition in the *P. nitida* and *D. cotinifolia* forests. The activity of acid phospomonoesterase (AP) in the decomposition of fresh leaf litter was lower in the *P. nitida* forest and higher in the *D. cotinifolia* forest compared to that in control. The most dominant soil bacteria were *Proteobacteria* in the *P. nitida* forest and were *Actinobacteria* and *Proteobacteria* in the *D. cotinifolia* forest. Shannon, Chao1, ACE and PD indexes in the mixed-litter decomposition of fresh leaf and semi-decomposition litter were higher than that in control in *P. nitida* forest. There were insignificant differences in observed species and indexes of Chao1, ACE and PD between litter treatments in the *D. cotinifolia* forest. Richness of mixed-litter significantly affected mass loss, soil enzyme activity and microbial diversity in the *P. nitida* forest. Litter N concentration and the presence of fresh leaf litter were significantly correlated with the mass loss and soil enzyme activity in the *P. nitida* and *D. cotinifolia* forests. These results indicated that the presence of fresh leaf litter showed a non-negligible influence on mixed-litter decomposition and soil enzyme activity, which might be partly explained by litter initial quality in the *P. nitida* and *D. cotinifolia* forests.

## Introduction

There is an important role of litter decomposition in the nutrient budget of forest ecosystems (Krishna and Mohan, 2017). Litter decomposition might enhance soil fertility because of the transfer and storage of carbon into humic substances and mineral nutrients during litter decomposition (Berg and McClaugherty, 2014). Therefore, a better understanding of the relative contribution of litter decomposition is important in predicting ecosystem processes and functions (Aerts, 2010). In biodiverse ecosystems, litter is often mixed and decomposes as a group of different plant species rather than decomposes separately from component species (Gartner and Cardon, 2004). As a results, the relationship between biodiversity and litter decomposition has attracted much attention in recent years, and the results were not consistent (Beaumelle et al., 2020; Mori et al., 2020; Kou et al., 2020; Boyero et al., 2021). Some studies found that the decomposition rate of litter composed of several species was significantly higher than that of a single species, while some studies found higher species diversity (richness and composition) had negative effects on litter decomposition (Gessner et al., 2010; Handa et al., 2014). In addition, some studies showed that the initial chemical traits of the composite litter rather than species richness are the most fundamental factor underlying litter mixing effect (Hättenschwiler et al., 2005; Handa et al., 2014; Santonja et al., 2017). These different results were received from various environments, which implies that the interrelation between species diversity and litter decomposition need to be considered in a specific context, including ecosystem type (Mori et al., 2020; Boyero et al., 2021; Luan et al., 2021).

Previous studies have shown that there are three stages of decomposition processes of litter (Berg and McClaugherty, 2014). First, the leaching process is the main reason for the mass loss of litter. Then, the soluble and nonlignified components (cellulose and hemicellulose) of litter begin to degrade (Heim and Frey, 2004). Finally, the decomposition of lignified components occurs, which is mainly regulated by litter chemistry (Zhou et al., 2015). Considering the important influences of litter decomposition on soil fertility due to the transfer and storage of carbon into humic substances and mineral nutrients during litter decomposition in forest ecosystems, it is necessary to explore the influence of the decomposition of semi-decomposed litter on the nutrient cycling in forest ecosystems. To date, innumerable studies have assessed litter decomposition progress using fresh leaf litter, mainly because fresh leaf litter accounts for 70% of the annual litter generated (Robertson and Paul, 1999). Unfortunately, the effect of semi-decomposed litter on nutrient cycling in forest ecosystems has been neglected. Furthermore, in the subtropical evergreen broadleaf forest of Southwest China, fresh leaf litter and semi-decomposed leaf litter are usually mixed due to the perennial warm and humid climate (Qiao et al., 2014; Wang et al., 2016). However, research on the mixed-litter decomposition progress of fresh leaf and semi-decomposed leaf is scarce.

The root is an important vital organ of plants because it can support the acquisition of nutrients and water by aboveground plants, which is essential to plant growth. Previous studies have reported that the degradation of root litter also plays an important role in the global carbon (C) cycle and supports nutrients for plant growth to maintain the ecosystem *via* primary production, especially nitrogen (N) and phosphorus (P) (Parton et al., 2007; Gessner et al., 2010). However, the decomposition progress of root litter and its influence on soil C and N cycling have been frequently overlooked, although there is increasing interest in root litter degradation (Zhang et al., 2008; Freschet et al., 2013). Considering that fine roots (<2 mm in diameter) are the most active physiological components of tree roots (McCormack et al., 2015; Wang et al., 2016; Cai et al., 2019), few studies have focused on the effects of the decomposition of single fine root on the mass loss of root litter and soil C and N cycling (Gill and Burke, 2002; Vivanco and Austin, 2006; Smith et al., 2014). In the present study, the natural evergreen broadleaf forest, which is located in a typical Danxia landform in southwest China, are usually shallow-rooted plants due to the shallow soil. Plant fine roots in the natural evergreen broadleaf forest are frequently intermingled with above-ground litter (fresh leaf litter and semi-decomposed litter) and decomposed in mixed-litter. Thus, a better understanding of the mixed-litter decomposition of fine root and above-ground litter (fresh leaf litter and semi-decomposed litter) and its effect on soil microbial communities and functions is imperative in the evergreen broadleaf forest of southwestern China.

Microbial communities and functions of soil might change due to their substrate preferences and strategies of nutrient acquisition during litter decomposition (Goldfarb et al., 2011). During litter decomposition, the decomposition rate and soil nutrient cycling are altered by the extracellular enzymes secreted by soil microorganisms, which then alter the biogeochemical properties of ecosystems. Numerous studies have found that soil enzymatic activities might exhibit positive, neutral, or negative responses to litter decomposition (Kotroczó et al., 2014; Zhao et al., 2017; Liu et al., 2021). Additionally, several studies assessed the influences of litter decomposition on the soil microbial community, and the results showed that the soil microbial community structure was closely related to plant substrate (such as litter) availability (Yang et al., 2021). Furthermore, according to studies of mono-specific litter decomposition, litters with initial good-quality (low C:N or lignin:N ratios) are usually expected to promote the activities of soil microorganisms leading to a faster decomposition rate than litters with initial low quality (Berg, 2014; Setiawan et al., 2016). Limited studies have assessed the influences of mixed litter decomposition on the soil microbial community and function, and the results showed that bacterial abundance was found to be higher in mixed litter than in mono-specific litter (Zhang et al., 2019). The responses of the composition and diversity of microbial communities to mixed litter decomposition were different from those to mono-specific litter decomposition (Aneja et al., 2006; Kubartová et al., 2009; Santonja et al., 2017; Pereira et al., 2019).

The evergreen broadleaf forests are an important part of the vertical band spectrum of evergreen broad-leaved forests in southwestern China. These forests not only contain abundant rare species but also play an important role in carbon pools. In these forests of China, understory species (including some dwarf shrubs and herbs) are usually shallow-rooted plants, and their roots are mostly concentrated in the litter layer and the soil surface (Qiao et al., 2014; Wang et al., 2016). In recent years, these forests have been deforested and destroyed for land reclamation, resulting in different degrees of destruction of soil fertility. To restore soil fertility in the evergreen broadleaf forest, understanding the mixed-litter decomposition of fine root and above-ground litter (fresh leaf litter and semi-decomposed litter) and its effect on soil microbial communities and functions is imperative. Hence, for the aims to evaluate the effects of mixed-litter of fresh leaf litter, semi-decomposed litter and fine root on litter mass loss, soil enzyme activity and microbial community in the evergreen broadleaf forest, a laboratory microcosm experiment with a full factorial design containing 8 possible litter combinations of fresh leaf litter, semi-decomposed litter and fine root in two forests of *Parakmeria nitida* and *Dayaoshania cotinifolia*, which are unique protective species and dominant species in the evergreen broadleaf forest, was performed in this study. We hypothesize that (1) the litter mixing was beneficial to the decomposition of fresh leaf litter, semi-decomposed litter and fine root; (2) the responses of soil enzyme activity and microbial diversity to the litter decomposition vary in different litter treatments; (3) the effects of litter mixing on litter mass loss, soil enzyme activity and microbial diversity depended primarily on the composition of mixed-litter rather than the richness of mixed-litter.

## Materials and methods

### Study area

**Figure 1:**
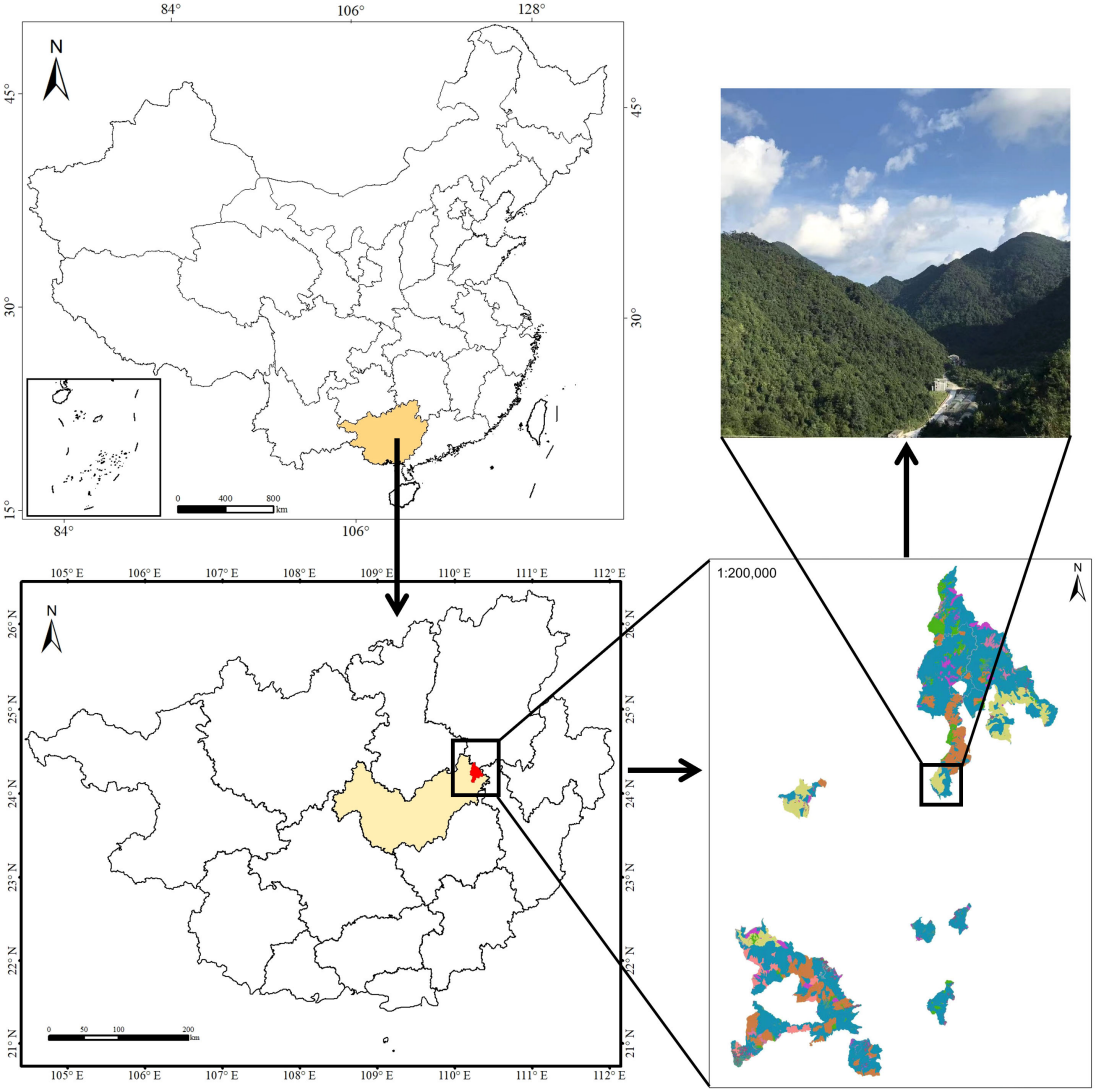
Map of Dayaoshan National Nature Reserve where the present study was carried out.

The study area was located in an evergreen broadleaf karst forest at the Dayaoshan National Nature Reserve (109°50′E~110°27′E, 23°40′N~24°28′N; total area 25594.7 hm^2^) in Guangxi Zhuang Autonomous Region southwest China (**Figure 1**). The site is located in the transition zone from south subtropical to mid-subtropical. The annual average sunshine hours are 1268.6 hours. The average temperature is 17.0°C, the annual extreme maximum temperature is 32.6°C, and the annual extreme minimum temperature is -5.6°C. The average annual rainfall is 1824.0 mm, and the annual average evaporation is 1203.0 mm. There are arbor layer and herb layer in the evergreen broadleaf forest of the Dayaoshan National Nature Reserve. Dominant species in the arbor layer and herb layer include *Castanopsis fabri* Hance, *Cyclobalanopsis jenseniana*, C. *fordii* Hance, *Meliosma squamulata* Hance, *Parakmeria nitida*, *Pleioblastus amarus* (Keng) Keng f., *Erythroxylum sinensis* C. Y. Wu, *Antidesma japonicum* Sieb. et Zucc, *Maesa japonica* (Thunb.) Moritzi. ex Zoll., *Sarcandra glabra* (Thunb.) Nakai, *Dictyocline sagittifolia* Ching, *Plagiogyria distinctissima* Ching, *Phyllagathis cavaleriei* (H. Lév.&Vaniot) Guillaumin, *Dayaoshania cotinifolia* W. T. Wang.

### Material collection and preparation

Fresh leaf litter, semi-decomposed litter, fine root and soil (0–20 cm) used in our study were collected in the *Parakmeria nitida* and *Dayaoshania cotinifolia* forests in the evergreen broadleaf forest. *P. nitida* and *D. cotinifolia* are unique protective plant in the Dayao Mountain, and they are the dominant species in the arbor layer and the herb layer, respectively. Fresh leaf litter was collected by a nylon net (2 mm mesh; 1 m × 1 m) from June to October 2019. Under the nylon net, semi-decomposed litter, fine root and soil (0-20 cm) were collected in October 2019. After collecting semi-decomposed litter from the soil surface, eight soil samples under nylon net were randomly collected by using a soil corer with an inner diameter of 4.5 cm, and then they were pooled together to give one composite sample. After sieved (< 2 mm), soil and roots were collected and then stored at 4°C until incubation. There were total 8 nylon nets in the *P. nitida* and *D. cotinifolia* forests (4 replicates × 2 forest types). Then, the fresh leaf litter, semi-decomposed litter, fine root and soil collected from each 4 nylon nets in the two forests were mixed, respectively. Before stored, soil water-holding capacity was calculated as:

$$\text{Water holding capacity }\left(\%\right)=\frac{\text{Mass wet-Mass dry}}{\text{Mass dry}}\times 100$$

made all possible 4 combinations of three types of litter: fresh leaf litter + semi-decomposed litter (FS); fresh leaf litter + fine root (FR); semi-decomposed litter + fine root (SR) and fresh leaf litter + semi-decomposed litter + fine root (FSR). Decomposition experiments were carried out in the same way as for no litter decomposition (CK) and individual species decomposition (single fresh leaf litter (FL); single semi-decomposed litter (SD); single fine root (RT)) using a laboratory microcosm (Mao et al., 2015). Filling plastic cups (7 cm in diameter and 9 cm high) with fresh soil (approximately equal to 80 g oven-dried soil), a total of 1 g of single litter or litter mixtures (with an equal mass proportion) was placed on the soil surface of microcosms. Thus, there were total 64 plastic cups (8 treatments × 4 replicates × 2 forest types). The cups were covered with a perforated adherent film to reduce humidity loss while allowing gaseous exchanges, and soil moisture was adjusted to 60% initial water holding capacity throughout the incubation period. Soil was harvested after 180 days of incubation (25°C), and then the soil was divided into three sub-samples. One sub-sample was air-dried at 25°C for chemical analysis, one sub-sample was stored at 4°C for enzyme analysis and the third sub-sample was freeze-dried prior to storage at -20°C for microbial community analysis.

### Chemical analyses and soil enzyme activity

After the fresh leaf litter, semi-decomposed litter and fine root were milled (<0.25 mm), the initial concentrations of total C and total N of fresh leaf litter, semi-decomposed litter, fine root and soil SOC concentration were measured using the K_2_Cr_2_O_7_–H_2_SO_4_ wet oxidation method of Walkley and Black (Nelson and Sommers, 1982). The total N concentration was determined with a continuous-flow autoanalyzer (AutoAnalyzer III, Bran+Luebbe GmbH, Germany) after the litter and soil samples were digested with H_2_SO_4_. The C/N ratio of litter was calculated from the values of total C concentration and total N concentration. The C/N ratio of the soil was calculated from the SOC concentration and total N concentration.

The activities of β-glucosidase (BG), N-acetyl-β-D-glucosaminidase (NAG), leucine aminopeptidase (LAP), and acid phospomonoesterase (AP) were measured at 180 incubation days according to the modified method of Tabatabai (1994) and Sinsabaugh et al. (1999). Briefly, 1.0 g of field-moist soil samples were suspended and homogenized in 0.25 mL of sodium acetate buffer, pH 6.0 (approxi-mate to the general acidic conditions of the soils), using a Midea blender for 1 min. For the potential activity of BG (EC 3.2.1.21), after incubation at 37°C for 1 h, 1 ml of 0.5 M CaCl_2_ and 4 ml of 0.1 M Tris buffer (pH = 12) were added to stop the reaction. Following filtration, the absorbance was measured at 400 nm using a spectrophotometer (UV 2550; Shimadzu, Japan). The potential activity of NAG (EC 3.2.1.30) was estimated in a similar way as the potential activity of BG, except that an acetate buffer (pH = 5.5) was used as the buffer solution, *p*-nitrophenyl-N-acetyl-β-D-glucosaminide was used as the substrate, and 1 ml of 0.5 M CaCl_2_ and 4 ml of 0.5 M NaOH were used to terminate the reaction. The potential activity of LAP was determined from the *p*-nitroaniline concentration released when the soil was incubated with leucine-*p*-nitroanilide in a Tris buffer. The potential activity of AP (EC 3.1.3.2) was measured with *p*-nitrophenyl phosphate as the substrate in a modified universal buffer of pH 6.5 after incubation for 1 h at 37°C. All values were determined in triplicate for each soil.

### Microbial community analysis

DNA extraction from the soil samples and Illumina MiSeq sequencing were submitted to Novogene Bioinformatics Technology Co., Ltd. (Tianjin, China) for Illumina MiSeq sequencing according to standard protocols. The extraction of soil DNA was performed with the Power MaxH Soil DNA Isolation Kit (MO BIO Laboratories, Inc., USA) according to the manufacturer’s operating instructions for bacteria. DNA concentration and purity were monitored by electrophoresis on a 1% agarose gel (NanoDrop Technologies, Wilmington, USA). Amplification and barcoded pyrosequencing of the 16S rRNA gene were conducted according to a previously described instruction (Sengupta and Dick, 2017). The fusion primer pair of 515F (GTGCCAGCMGCCGCGG) and 907R (CCGTCAATTCMTTTRAGTTT) was used to amplify the hypervariable V4 and V5 regions in triplicate. The purified DNA was generated from a sequencing library on the Illumina HiSeq 2500 platform (Illumina, San Diego, CA, USA).

Data obtained from Illumina sequencing were handled by separating primers and adapters from the reads, and a reading of approximately 250 bp was obtained. Sequences with >97% similarity were clustered into one operational taxonomic unit (OTU) using the UPARSE algorithm (v. 7.0.1001 http://drive5.com/usearch/manual/singletons.html). The α-diversity of the bacterial community, including observed OTU numbers, Shannon index, Simpson index, Chao1 index, ACE index, and PD index, was determined using Quantitative Insights Into Bacterial Ecology (QIIME, v. 1.7.0).

### Statistical analysis

The mass losses of fresh litter, semi-decomposed litter and fine root in monoculture and mixtures were calculated as follows:


$$Mass\;loss\;(\%)=\frac{m_{0\;}-m_{t}}{m_{t}}\times 100$$

where m_0_ and m_t_ are the dry weights of fresh litter, semi-decomposed litter and fine root in monoculture and mixtures at initial time and 180 days of incubation time, respectively.

The differences in total C concentration, total N concentration and C/N ratio among fresh leaf litter, semi-decomposed litter, fine root and soil were evaluated by one-way analysis of variance (ANOVA). The differences in litter mass loss, soil enzyme activities and microbial diversity among the 8 litter decomposition treatments were evaluated by one-way analysis of variance (ANOVA). Duncan multiple range tests were applied for pairwise comparison of the means (SPSS 16.0). The unweighted pair-group method with arithmetic mean (UPGMA) was used to analyze the similarity of soil microorganisms during litter decomposition. An analysis of similarity (ANOSIM) routine was used to evaluate the effects of the presence and absence of a particular species and the richness of mixed-litter on the soil enzyme activities and microbial diversity exposed to the decomposition of mixed-litter. The homogeneity of within-group variances was tested before conducting the ANOSIM using the betadisper function in the “vegan” package in R. To assess how the differences in litter combinations affect soil enzyme activities and microbial diversity, the principal component analysis (PCA) were evaluated in the present study. The values of soil enzyme activities and microbial diversity were log-transformed (using the “standardize species” option in Canoco 5.0) before unconstrained PCA. Redundancy analysis (RDA) was used to test specific hypotheses about the relationship between litter chemical traits, litter mass loss, soil enzyme activities and microbial diversity (the values were log-transformed using the “standardize species” option in Canoco 5.0). Significance was based on permutation test using 999 permutations and a split-plot design (Lepš and Šmilauer, 2003).

## Results

### Litter mass loss

Initial chemical traits differed substantially among the fresh leaf litter, semi-decomposed litter, fine root and soil (**Table 1**). The total C concentration and C/N ratio of fresh leaf litter in the *P. nitida* forest were higher than those of semi-decomposed litter, fine root and soil. The total C and N concentrations and C/N ratio in the soil were lower than those in the fresh leaf litter, semi-decomposed litter and fine root in the *P. nitida* and *D. cotinifolia* forests. There was an insignificant difference in the total C concentration between fresh leaf litter and semi-decomposed litter in the *D. cotinifolia* forest. The total N concentration in the semi-decomposed litter was higher than that in the fresh leaf litter, fine root and soil in the *D. cotinifolia* forest. The C/N ratio in the soil was lower than that in the fresh leaf litter, semi-decomposed litter and fine root in the *D. cotinifolia* forest.

**Table 1:** Initial chemical traits ( ± SE) of the fresh leaf litter, semi-decomposed litter, fine root and soil of *Parakmeria nitida* and *Dayaoshania cotinifolia* forest.

	Litter type	Total C (%)	Total N (%)	C/N
*Parakmeria nitida*	Fresh leaf litter	41.9 (1.1) ^d^	1.5 (0.1) ^c^	28.4 (0.3) ^d^
	Semi-decomposed litter	9.1 (0.4) ^b^	0.4 (0.0) ^b^	26.0 (2.3) ^c^
	Fine root	38.5 (1.5) ^c^	1.7 (0.1) ^d^	22.9 (0.2) ^b^
	Soil	2.4 (0.1) ^a^	0.2 (0.0) ^a^	13.3 (0.8) ^a^
*Dayaoshania cotinifolia*	Fresh leaf litter	40.2 (0.2) ^c^	1.7 (0.0) ^b^	24.0 (0.2) ^d^
	Semi-decomposed litter	42.0 (0.4) ^c^	2.0 (0.0) ^c^	20.7 (0.2) ^b^
	Fine root	36.7 (2.8) ^b^	1.7 (0.1) ^b^	22.1 (0.1) ^c^
	Soil	22.2 (1.8) ^a^	1.4 (0.0) ^a^	15.4 (1.8) ^a^

Lowercase letters indicate statistical difference among the three litter and soil according to Duncan test (P< 0.05).

The mass loss of semi-decomposed litter was significantly higher in the *P. nitida* forest and lower in *D. cotinifolia* forest than the mass loss of single litter of fresh leaf and fine root (**Table 2**). In the *P. nitida* forest, the mass loss of fresh leaf litter in the FSR treatment was faster than that in the other three treatments (**Table 2**). There were insignificant differences in the mass loss of semi-decomposed litter between SD treatment and FS treatment and between SR treatment and FSR treatment. The mass loss of fine root in the *P. nitida* forest was lower in the RT treatment and higher in FSR treatment compared to the other three treatments. For the *D. cotinifolia* forest, the mass loss of fresh leaf litter and fine root in the treatment of single litter decomposition was higher than that in the treatment of mixed-litter decomposition (**Table 2**). There were insignificant differences in the mass loss of semi-decomposed litter between SD treatment and SR treatment, and between FS treatment and FSR treatment.

**Table 2:** Mass loss (± SE) of the fresh leaf litter, semi-decomposed litter and fine root in the litter decomposition treatments.

	Litter combinations	Mass loss (%)	
		*Parakmeria nitida*	*Dayaoshania cotinifolia*
Fresh leaf litter	Fresh leaf litter	21.0 (1.6) ^bB^	42.9 (2.4) ^dC^
	Fresh leaf litter + Semi-decomposed litter	13.5 (1.9) ^aA^	13.0 (1.9) ^aA^
	Fresh leaf litter + Fine root	14.7 (2.9) ^aB^	23.2 (1.8) ^bA^
	Fresh leaf litter + Semi-decomposed litter + Fine root	33.3 (5.6) ^cC^	31.0 (2.4) ^cB^
Semi-decomposed litter	Semi-decomposed litter	26.4 (1.4) ^bC^	24.5 (2.0) ^aA^
	Fresh leaf litter + Semi-decomposed litter	25.0 (1.9) ^bB^	36.4 (3.0) ^bB^
	Semi-decomposed litter + Fine root	11.1 (3.8) ^aA^	22.9 (2.1) ^aA^
	Fresh leaf litter + Semi-decomposed litter + Fine root	11.1 (4.8) ^aA^	34.0 (2.0) ^bB^
Fine root	Fine root	11.1(3.8) ^aA^	28.1 (1.0) ^cB^
	Fresh leaf litter + Fine root	13.2 (2.7) ^bA^	13.0 (4.3) ^aA^
	Semi-decomposed litter + Fine root	14.7 (2.9) ^cB^	22.0 (2.0) ^bA^
	Fresh leaf litter + Semi-decomposed litter + Fine root	19.4 (4.8) ^dB^	22.7 (2.3) ^bA^

Lowercase letters indicate statistical difference among the four litter treatments according to Duncan test (P< 0.05).

Uppercase letters indicate statistical difference among the 3 litter types according to Duncan test (P< 0.05).

The mass loss of mixed-litter was significantly affected by the richness and the presence of fresh leaf litter and semi-decomposed litter in the *P. nitida* forest and by the presence of fresh leaf litter and fine root in the *D. cotinifolia* forest (**Table 3**). The RDA results showed that litter mass loss was significantly explained by the total C and N concentrations and C/N ratio of litter in the *P. nitida* and *D. cotinifolia* forests (29.0%, 21.9%, 90.5%, 91.1%, 41.8% and 88.0%, respectively, **Table 4**).

**Table 3:** Analysis of similarity (ANOSIM) testing the effects of the presence of fresh leaf litter, semi-decomposed litter, fine root and richness of mixed-litter on mass loss, soil enzyme activity and microbial diversity in the *Parakmeria nitida* and *Dayaoshania cotinifolia* forests.

		Mass loss		Enzyme activity		Microbial diversity	
		R	*P*	R	*P*	R	*P*
*Parakmeria nitida*	Presence of fresh leaf litter	**0.2**	**0.028**	**0.1**	**0.025**	-0.1	0.919
	Presence of semi-decomposed litter	**0.3**	**0.003**	0.0	0.233	0.0	0.215
	Presence of fine root	0.1	0.108	**0.2**	**0.022**	0.0	0.755
	Richness	**0.1**	**0.073**	**0.2**	**0.047**	**0.2**	**0.040**
*Dayaoshania cotinifolia*	Presence of fresh leaf litter	**0.1**	**0.074**	**0.2**	**0.013**	0.0	0.511
	Presence of semi-decomposed litter	-0.1	0.816	0.0	0.361	0.0	0.308
	Presence of fine root	**0.2**	**0.009**	0.0	0.651	0.0	0.865
	Richness	0.1	0.069	0.4	0.004	-0.1	0.735

R, a ratio between within-group and between-group dissimilarities. The significant P values are displayed in bold form (P < 0.05).

**Table 4:** Redundancy analysis (RDA) showing the influences of litter chemical traits and mass loss on the responses of soil enzyme activity and microbial diversity to the mixed-litter decomposition in *Parakmeria nitida* and *Dayaoshania cotinifolia* forests.

		Mass loss			Enzyme activity			Microbial diversity		
		Explain (%)	*P*	F	Explain (%)	*P*	F	Explain (%)	*P*	F
*Parakmeria nitida*	C	**29.0**	**0.002**	**2.5**	**37.7**	**0.002**	**3.6**	**21.3**	**0.002**	**1.6**
	N	**21.9**	**0.002**	**1.7**	**40.4**	**0.002**	**4.1**	**27.7**	**0.002**	**2.3**
	C/N	**90.5**	**0.002**	**56.9**	18.3	0.260	1.3	7.5	0.732	0.5
	Mass loss	–	–	–	5.5	0.604	0.3	2.2	0.832	0.1
*Dayaoshania cotinifolia*	C	**91.1**	**0.002**	**61.3**	6.7	0.748	0.4	**30**	**0.002**	**2.6**
	N	**41.8**	**0.002**	**4.3**	**18.7**	**0.052**	**1.4**	**42.8**	**0.002**	**4.5**
	C/N	**88.0**	**0.002**	**44.1**	4.9	0.742	0.3	17.1	0.260	1.2
	Mass loss	–	–	–	2.9	0.912	0.2	2.5	0.822	0.2

Explained variance is based on the sum of all canonical eigenvalues. P-values are based on a Monte-Carlo permutation test with 999 permutations, and restricted for split-plot design. Significant differences were labeled with bold. R, a ratio between within-group and between-group dissimilarities.

### Soil enzyme activity and microbial community

**Figure 2:**
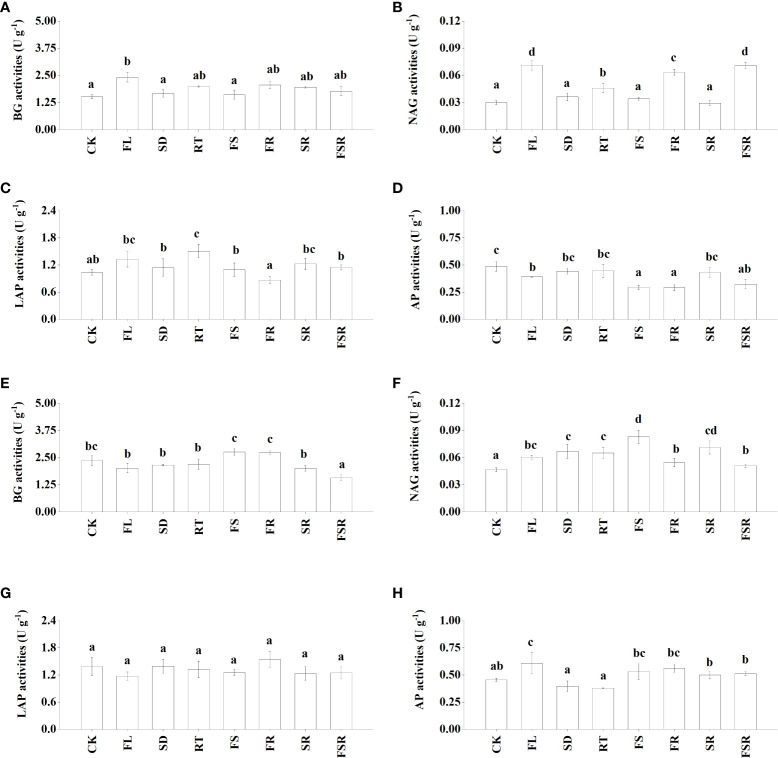
The responses of soil enzyme activities to the litter decomposition in *Parakmeria nitida* **(A–D)** and *Dayaoshania cotinifolia* **(E–H)** forests. Different letters above the bars represent significant differences from Duncan multiple comparisons among 8 litter treatments (*P*<0.05). CK, no litter decomposition; FL, the decomposition of fresh leaf litter; SD, the decomposition of semi-decomposed litter; RT, the decomposition of fine root; FS, the decomposition of fresh leaf litter + semi-decomposed litter; FR, the decomposition of fresh leaf litter + fine root; SR, the decomposition of semi-decomposed litter + fine root; FSR, the decomposition of fresh leaf litter + semi-decomposed litter + fine root; BG, β-glucosidase; NAG, N-acetyl-β-D-glucosaminidase; LAP, leucine aminopeptidase; AP, acid phospomonoesterase.

**Figure 3:**
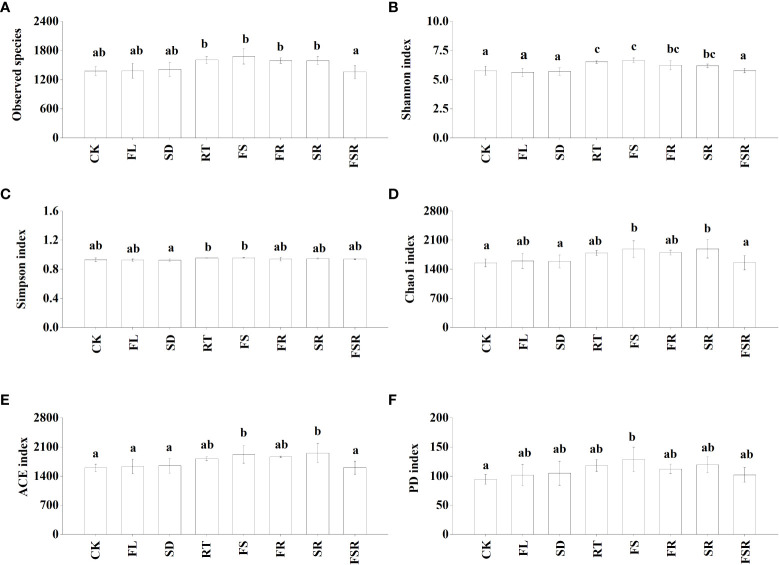
The responses of alpha diversity index of soil bacteria to the litter decomposition in *Parakmeria nitida* forest **(A–F)**. Different letters above the bars represent significant differences from Duncan multiple comparisons among 8 litter treatments (*P*<0.05). CK, no litter decomposition; FL, the decomposition of fresh leaf litter; SD, the decomposition of semi-decomposed litter; RT, the decomposition of fine root; FS, the decomposition of fresh leaf litter + semi-decomposed litter; FR, the decomposition of fresh leaf litter + fine root; SR, the decomposition of semi-decomposed litter + fine root; FSR, the decomposition of fresh leaf litter + semi-decomposed litter + fine root.

The activities of four extracellular enzymes participating in C-cycling (β-glucosidase; BG), N-cycling (N-acetyl-β-D-glucosaminidase, NAG; leucine aminopeptidase, LAP), and P-cycling (acid phospomonoesterase; AP) were measured in the present study. In the *P. nitida* forest, the BG activity was significantly higher in the FL treatment compared to that in CK treatment (**Figure 2A**). The NAG and LAP activities were higher in the RT treatment than that in the CK treatment (**Figures 2B, C**). The decomposition of two mixed-litter of fresh leaf and fine root and three mixed-litter of fresh leaf, semi-decomposed litter and fine root significantly increased soil NAG activity (**Figure 2B**). The soil AP activities were lower in the treatment of FL, FS, FR and FSR compared to that in the CK treatment (**Figure 2D**). In the *D. cotinifolia* forest, the decomposition of the three mixed-litter significantly decreased the soil BG activity (**Figure 2E**). The soil NAG activities were significantly higher in litter treatments than that in the CK treatment (**Figure 2F**). There were insignificant differences in soil LAP activity between the control and litter treatments (**Figure 2G**). Generally, the decomposition of mixed-litter showed insignificant effects on soil AP activity (**Figure 2H**). And the soil AP activity was significantly higher in the FL treatment compared to that in the CK treatment. The observed species were significantly lower in the FSR than that in the treatment of RT, FS, FR and SR in the *P. nitida* forest (**Figure 3A**).

**Figure 4:**
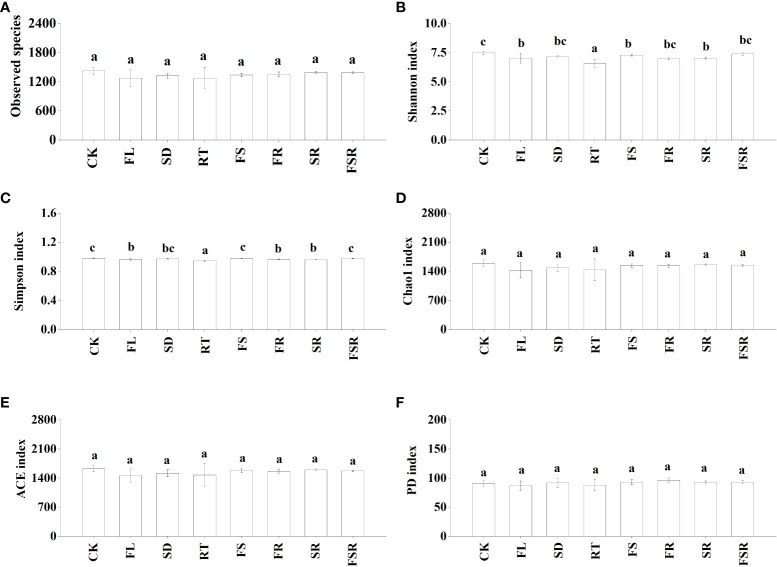
The responses of alpha diversity index of soil bacteria to the litter decomposition in *Dayaoshania cotinifolia* forest **(A–F)**. Different letters above the bars represent significant differences from Duncan multiple comparisons among 8 litter treatments (*P*<0.05). CK, no litter decomposition; FL, the decomposition of fresh leaf litter; SD, the decomposition of semi-decomposed litter; RT, the decomposition of fine root; FS, the decomposition of fresh leaf litter + semi-decomposed litter; FR, the decomposition of fresh leaf litter + fine root; SR, the decomposition of semi-decomposed litter + fine root; FSR, the decomposition of fresh leaf litter + semi-decomposed litter + fine root.

The Shannon index were significantly higher in the RT and FS treatments than that in the treatment of CK, FL, SD and FSR in the *P. nitida* forest (**Figure 3B**). The Simpson index was significantly higher in the RT and FS treatment than that in the SD treatment (**Figure 3C**). The indexes of Chao1, ACE and PD were significant higher in the FS treatment compared to that in the CK treatment (**Figures 3D** **–F**). In the *D. cotinifolia* forest, there were insignificant differences in the relative abundance of observed species, Chao1 index, ACE index and PD index between the control and litter treatments (**Figures 4A** **, D–F**). The Shannon index and Simpson index were significantly lower in the treatments of RT, FL and SR compared to that in the CK treatment (**Figure 4B, C**).

The richness of the mixed-litter had significant effects on the soil enzyme activity and microbial diversity in the *P. nitida* forest (**Table 3**). The soil enzyme activity was significantly affected by the presence of fresh leaf litter and fine root in the *P. nitida* forest, and by the presence of fresh leaf litter in the *D. cotinifolia* forest. The richness of mixed-litter rather than the presence of fresh leaf litter, semi-decomposed litter and fine root showed significant effects on soil microbial diversity in the *P. nitida* forest. However, the soil microbial diversity was insignificantly affected by the richness mixed-litter and the presence of fresh leaf litter, semi-decomposed litter and fine root in the *D. cotinifolia* forest. According to the results of RDA, soil enzyme activity and microbial diversity were significantly explained by the total C and N concentrations of litter in the *P. nitida* forest, and were significantly explained by the total N concentration of litter in the *D. cotinifolia* forest (37.7%, 40.4%, 21.3%, 27.7%, 18.7% and 42.8%, respectively, **Table 4**).

**Figure 5:**
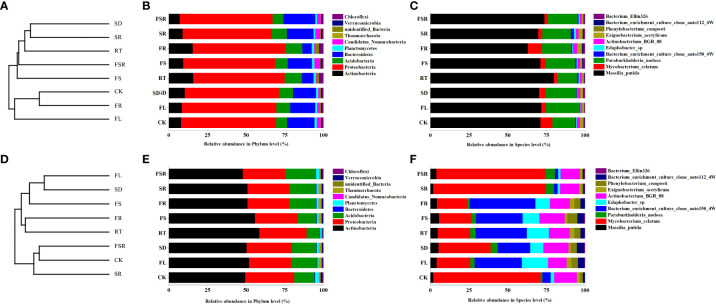
UPGMA clustering tree and relative abundance of soil bacteria exposed to the litter decomposition in *Parakmeria nitida* **(A–C)** and *Dayaoshania cotinifolia* **(D–F)** forests. CK, no litter decomposition; FL, the decomposition of fresh leaf litter; SD, the decomposition of semi-decomposed litter; RT, the decomposition of fine root; FS, the decomposition of fresh leaf litter + semi-decomposed litter; FR, the decomposition of fresh leaf litter + fine root; SR, the decomposition of semi-decomposed litter + fine root; FSR, the decomposition of fresh leaf litter + semi-decomposed litter + fine root.

The most dominant soil bacteria in the *P. nitida* forest and *D. cotinifolia* forest were different (**Figure 5**). In the *P. nitida* forest, the most dominant soil bacteria were *Proteobacteria*. In the *D. cotinifolia* forest, the most dominant soil bacteria were both *Actinobacteria* and *Proteobacteria*. At the phylum level, there was insignificantly differences in the relative abundance of *Proteobacteria* between the 8 litter decompositions, while, at the species level, the relative abundance of *Massilia_putida* was higher in the RT treatment compared to that in the other litter treatment in the *P. nitida* forest (**Figures 5B, C**). In the *D. cotinifolia* forest, at the phylum level, *Actinobacteria* and *Proteobacteria* exhibited higher relative abundance in the single decomposition of fine root compared to that in the other litter decomposition (**Figure 5E**). At the species level, the relative abundance of *Massilia_putida* and *Mycobacterium_celatum* was significantly lower in the treatments of FL, SD, RT, FS and FR than that in the CK, SR and FSR treatments (**Figure 5F**). To evaluate the impact of the diversity of the soil microbial community during the decomposition of mixed-litter, UPGMA clustering was used in this study by the unweighted UniFrac distance matrix (**Figures 5A, D**). The present results found that the beta diversity of the soil was different among the 8 litter decomposition treatments and between the two plant species.

**Figure 6:**
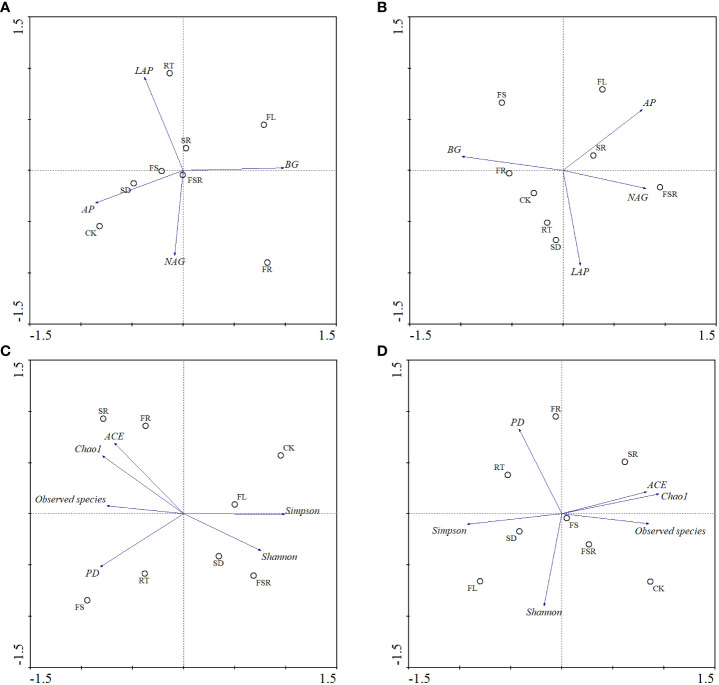
Bi-plots of principal component analysis (PCA) on the soil enzyme activities and microbial diversity exposed to the litter decomposition in *Parak meria nitida* **(A, C)** and *Dayaoshania cotinifolia* **(B, D)** forests. CK, no litter decomposition; FL, the decomposition of fresh leaf litter; SD, the decomposition of semi-decomposed litter; RT, the decomposition of fine root; FS, the decomposition of fresh leaf litter + semi-decomposed litter; FR, the decomposition of fresh leaf litter + fine root; SR, the decomposition of semi-decomposed litter + fine root; FSR, the decomposition of fresh leaf litter + semi-decomposed litter + fine root; BG, β-glucosidase; NAG, N-acetyl-β-D-glucosaminidase; LAP, leucine aminopeptidase; AP, acid phospomonoesterase; Shannon, Shannon index; Simpson, Simpson index; Chao1, Chao1 index; ACE, ACE index; PD, PD index.

According to the principal component analysis (PCA), the soil BG and AP activities strongly contributed to the PC1, and the activities of LAP and NAG strongly relationship with PC2 in the *P. nitida* forest (**Figure 6A**). The Simpson index, relative abundance of observed species and FL treatment strongly contributed to the PC1 in the *P. nitida* forest (**Figure 6C**). The FR, RT and SD treatments strongly clustered along PC2 in the *P. nitida* forest. For the *D. cotinifolia* forest, the soil BG activity, NAG activity and the treatments of FR, SR and FSR strongly contributed to the PC1, and soil LAP activity and the treatments of SD and RT strongly clustered along PC2 (**Figure 6B**). The relative abundance of observed species, ACE index, Chao1 index, Simpson index and the SD treatment strongly clustered along PC1, and Shannon index and FSR treatment strongly contributed to the PC2 in the *D. cotinifolia* forest (**Figure 6D**).

## Discussion

In this study, the mass loss of fresh leaf litter was significantly higher than the mass loss of fine root during the decomposition of single litter in the *P. nitida* and *D. cotinifolia* forests, respectively. Similarly, previous results showed that leaf litter with rich N and P contents breaks down more easily than root litter with poor quality during single litter decomposition (Xing et al., 2019; Luo et al., 2020). In the present study, only the coexistence of semi-decomposed litter and fine root significantly increased the mass loss of fresh leaf litter in the *P. nitida* forest. The mass loss of fine root in the single litter decomposition was lower than that in the decomposition of two and three mixed-litter in the *P. nitida* forest. Furthermore, the presence of fresh leaf litter and fine root did not significantly increase the mass loss of the semi-decomposed litter. These results indicated that three litter mixing was beneficial to the decomposition of fresh leaf litter and fine root but not semi-decomposed litter in the *P. nitida* forest. In contrast, three litter mixing was not beneficial to the decomposition of fresh leaf litter and fine root but was conducive to the decomposition of semi-decomposed litter in *D. cotinifolia* forests. These results were different from our first hypothesis.

Soil enzymes are the primary drivers of plant litter degradation in most ecosystems (Koeck et al., 2014; de Carvalho Mendes et al., 2019). However, a clear link between soil enzyme activities and the decomposition of fresh leaf litter, semi-decomposed litter and fine root is often rare. In the present study, the changes in soil enzyme activity varied considerable in different litter treatments in the *P. nitida* and *D. cotinifolia* forests, which was consistent with our second hypothesis. In the *P. nitida* forest, the single decomposition of fresh leaf litter and fine root induced increases in N-acquiring enzyme (NAG and LAP) activity. This was consistent with results shown by Fang et al. (2018), who demonstrated that the addition of wheat residues increased soil BG activity and NAG activity. Furthermore, the decomposition of single fresh leaf litter, two mixed-litter of fresh leaf litter and semi-decomposed litter, two mixed-litter of fresh leaf litter and fine root and three mixed-litter were significantly decreased soil AP activity in the *P. nitida* forest. This result indicated that the addition of fresh leaf litter, semi-decomposed litter and fine root was not conducive to soil P acquisition in the *P. nitida* forest. In contrast to the *P. nitida* forest, there were insignificant differences in soil BG activity among the control treatment, single litter decomposition and the decomposition of the two mixed-litter in the *D. cotinifolia* forest. The decomposition of three mixed-litter significantly induced decreases in BG activity. Thus, the addition of litter was not conducive to soil C acquisition in the *D. cotinifolia* forest. The decomposition of mixed-litter significantly increased the soil NAG activity compared to control, indicating that the litter addition was beneficial to soil N acquisition in the *D. cotinifolia* forest.

Previous study showed that copiotrophs and oligotrophs play an important role in the composition of the microbial community during litter decomposition (Sun et al., 2013). *Proteobacteria* and *Bacteroidetes* are the dominant bacterial groups in copiotrophic phyla, which have a stronger adaptability to soil nutrients (Větrovský and Baldrian, 2013; García-Palacios et al., 2016; Dong et al., 2021). The nutrients (ammonia and methane) produced by the decomposition of organic matter were mainly utilized by *Proteobacteria* during litter decomposition (Lv et al., 2014). *Actinobacteria* and *Acidobacteria*, which are mainly involved in the decomposition of refractory compounds of litter (Lydell et al., 2004; Pankratov et al., 2011), are the dominant bacterial groups in oligotrophic phyla. Some studies found that litter decomposition significantly increased soil copiotrophic groups (Fierer et al., 2007; Chodak et al., 2014) and decreased the oligotrophic phyla of soil (Fierer et al., 2007). In this study, the most dominant soil bacteria were *Proteobacteria* in the *P. nitida* forest, while, in the *D. cotinifolia* forest, the most dominant soil bacteria were both *Actinobacteria* and *Proteobacteria*. Furthermore, in the *P. nitida* forest, the decomposition of single fine root and three mixed-litter significantly increased the relative abundance of *Massilia_putida* compared to the other litter decomposition. Therefore, the addition of single fine root and three mixed-litter favored the growth of *Proteobacteria* in the *P. nitida* forest. Differently, the single litter decomposition significantly decreased the relative abundance of *Massilia_putida* and *Mycobacterium_celatum* in the *D. cotinifolia* forest, indicating that the growth of *Actinobacteria* and *Proteobacteria* was inhibited by single litter addition.

Previous study found that the soil microbial diversity was reduced by litter decomposition in mixed forests (Hooper et al., 2000; Merloti et al., 2022). However, Nielsen et al. (2010) found that there has been no consistent conclusion on the effects of litter decomposition in mixed forests on the soil microbial community. Similarly, there were notable differences in how the soil microbial diversity responded to litter decomposition of fresh leaf litter, semi-decomposed litter and fine root in the present study, which was consistent with our second hypothesis. Generally, the present results found that soil bacterial evenness (Shannon and Simpson) were varied considerable in different composition of mixed-litter in the *P. nitida* and *D. cotinifolia* forests, and soil bacterial richness (Chao1 and ACE) in the *P. nitida* forest was significantly increased by the decomposition of the two mixed-litter of fresh leaf and semi-decomposed litter and two mixed-litter of semi-decomposed litter and fine root. Differently, soil bacterial richness (Chao1 and ACE) was insignificantly affected by litter decomposition in the *D. cotinifolia* forest. According to the RDA results in the present study, soil microbial diversity was significantly related to the litter C and N concentrations. Therefore, to a certain extent, the changes in soil microbial diversity was partly related to the initial C and N concentrations of litter in the *P. nitida* and *D. cotinifolia* forests (Zhao et al., 2016; Chen et al., 2021).

Furthermore, there are many studies examining how litter mixing from different plant species affects decomposition rate (Gartner and Cardon, 2004; Hättenschwiler et al., 2005). Species richness and species composition are often used to assess how species biodiversity influences the effects of species mixing on litter decomposition rate (Ball et al., 2008; De Marco et al., 2011; Mao et al., 2015). However, it remains unclear how decomposition progress responds to the mixed-litter of fresh leaf litter, semi-decomposed litter and fine root, especially how the decomposition rate and the soil microbes can be altered by changes in litter richness and litter composition. The present study found that the richness of mixed-litter and presence of fresh leaf significantly affected the litter mass loss and soil enzyme activity in the *P. nitida* forest. Differently, the presence of fresh leaf litter rather than richness of mixed-litter significantly affected litter mass loss and soil enzyme activity in the *D. cotinifolia* forest. These results were different from our third hypothesis. Recent studies have suggested that litter quality (initial chemical traits such as C, N and C/N ratio) could be a more important factor than other factors (such as climate) in controlling the decomposition rate and soil microbes during the single litter decomposition across different biomes worldwide (Hättenschwiler et al., 2005; Bradford et al., 2014; Berg et al., 2015; De Long et al., 2016). Similarly, the results of the present study showed that the initial C and N concentrations and C/N ratio of litter were significantly correlated with litter mass loss (explanations were 29.0%, 21.9%, 90.5%, 91.1%, 41.8% and 88.0%, respectively), and litter initial N concentration was significantly correlated with soil enzyme activity (explanations were 40.4% and 18.7%, respectively) in the *P. nitida* and *D. cotinifolia* forests. Therefore, the effects of litter mixing on litter mass loss depended primarily on the presence of fresh leaf litter which was mainly explained by initial litter C/N ratio in the *P. nitida* forest, and by the initial C concentration and C/N ratio of litter in the *D. cotinifolia* forest. Furthermore, the effects of litter mixing on soil enzyme activity also primarily depended on the presence of fresh leaf litter, which might be partly explained by the litter N concentration in the *P. nitida* and *D. cotinifolia* forests. Thus, the presence of fresh leaf litter showed a non-negligible influence on mixed-litter decomposition and soil enzyme activity, which might be partly explained by litter initial quality in the *P. nitida* and *D. cotinifolia* forests.

## Conclusions

In the present study, we analyzed the changes in litter mass loss, soil enzyme activity and soil microbial community during the mixed-litter decomposition of fresh leaf, semi-decomposed litter and fine root in the *P. nitida* and *D. cotinifolia* forests. We observed that three litter mixing was beneficial to the litter decomposition of fresh leaf and fine root but not semi-decomposed litter in the *P. nitida* forest, while it was conducive to the decomposition of semi-decomposition litter but not fresh leaf litter and fine root in the *D. cotinifolia* forest. The changes in soil enzyme activity and microbial diversity varied considerable in different litter treatments, and the changes in microbial diversity were partly related to the initial C and N concentrations of litter in the *P. nitida* and *D. cotinifolia* forests. The most dominant soil bacteria were *Proteobacteria* in the *P. nitida* forest, and were *Actinobacteria* and *Proteobacteria* in the *D. cotinifolia* forest. Furthermore, the effects of litter mixing on mass loss depended primarily on the presence of fresh leaf litter which was mainly explained by initial litter C/N ratio in the *P. nitida* forest, and by the initial C concentration and C/N ratio of litter in the *D. cotinifolia* forest. Similarly, the responses of soil enzyme activity to the litter mixing were significantly affected by the presence of fresh leaf litter, which might be partly explained by the litter N concentration in the *P. nitida* and *D. cotinifolia* forests. Thus, the presence of fresh leaf litter showed a non-negligible influence on mixed-litter decomposition and soil enzyme activity, which might be partly explained by litter initial quality in the *P. nitida* and *D. cotinifolia* forests. Finally, it should be pointed out that only initial C and N concentrations and C/N ratio of litter were used to evaluate the influence of litter quality on decomposition process in the present study. As litter decomposition is a complex process, nutrient transfer among component species of the litter mixture also might modify and change the decomposer community. Hence, to further understanding the mechanisms by which microbial processes facilitate litter decomposition, nutrient release and more number of chemical traits and physical traits should be considered in the further study.

## Data availability statement

The data presented in this study are deposited in the NCBI repository, accession number PRJNA903116.

## Author contributions

BM and TS: original draft preparation. BM, TS and TC: methodology. TC and SX: software. QX, FL, HS and JZ: sample collection. BM, SX and TS: writing— review and editing. All authors contributed to the article and approved the submitted version.
